# Measurement Along the Path of Unmanned Aerial Vehicles for Best Horizontal Dilution of Precision and Geometric Dilution of Precision

**DOI:** 10.3390/s25133901

**Published:** 2025-06-23

**Authors:** Yanwu Ding, Dan Shen, Khanh Pham, Genshe Chen

**Affiliations:** 1 Department of Electrical and Computer Engineering, Wichita State University, Wichita, KS 67260, USA; 2 Intelligent Fusion Technology, Germantown, MD 20874, USA; dshen@intfusiontech.com (D.S.); gchen@intfusiontech.com (G.C.); 3 Air Force Research Laboratory, Albuquerque, NM 87108, USA

**Keywords:** geometric dilution of precision, GDOP, HDOP, DOP, VDOP, geometric matrix, UAV, average GDOP, average HDOP

## Abstract

In the zenith-horizon placement for achieving minimum geometric dilution of precision (GDOP), one access node or sensor is positioned along the z-axis, while the remaining nodes are placed symmetrically on a three-dimensional (3D) cone. This configuration yields the minimum GDOP at the cone’s tip, which we term the designated min-GDOP point. However, in practical localization applications, the unknown node is not necessarily located at this designated min-GDOP point; instead, it may be situated anywhere within an area. As a result, evaluating localization accuracy across the entire area, rather than at a single point, is more relevant. Averaged horizontal dilution of precision (HDOP) and GDOP across the region provide more meaningful metrics for system-wide performance than values computed only at a specific location. Although many recent positioning applications leverage multiple unmanned aerial vehicles (UAVs), many established fixed sensor deployments predate the widespread adoption of UAVs. This paper proposes a novel approach with a single UAV working in conjunction with existing fixed access nodes for positioning. This approach offers improved adaptability for fixed infrastructure while circumventing the expense of establishing entirely new UAV systems, thus providing a valuable compromise. We investigate the criteria of average HDOP and GDOP over the given area. The objective is to determine optimal UAV positions along the flight path that minimize the average HDOP and/or GDOP across the area. Due to the analytical complexity, we employ numerical methods. Our simulation results demonstrate that minimizing average HDOP and GDOP often requires different UAV positions, depending on the number of access nodes and the size of the area. Consequently, achieving simultaneous minimization of both metrics with a single UAV trajectory is generally infeasible. When minimizing the average HDOP with a small number of access nodes, aligning the UAV’s XY-plane angle with those of the stationary nodes, offset by $60^{\circ }$, proves advantageous. This angular alignment becomes less critical as the number of access nodes increases. For scenarios where both HDOP and GDOP are important, UAV placement can be optimized by selecting appropriate trade-offs. Additionally, we quantify how increasing the number of access nodes improves the average HDOP and GDOP over the specified area.

## 1. Introduction

The metric of geometric dilution of precision (GDOP) is widely used for assessing the accuracy and reliability of position estimation. It directly characterizes the relationship between measurement observations and position estimation [1,2,3]. The accuracy of position estimation increases as GDOP decreases [2,4,5]. The GDOP was initially introduced for the geometric arrangement and selection of satellites for a receiver on Earth. It remains an important analysis method for quickly estimating the observability of satellites quantitatively and for selecting satellites to improve positioning accuracy in the Global Positioning System (GPS) and global navigation satellite systems (GNSSs) [6,7,8,9,10]. The main goal of the selection algorithm is to minimize the GDOP to improve positioning accuracy based on the pseudo-range measurement [11,12,13,14].

The GDOP is related to the horizontal dilution of precision (HDOP) and vertical dilution of precision (VDOP) [15,16,17]. The importance of HDOP and VDOP varies; HDOP is more relevant when the unknown source or target is at a fixed height where horizontal accuracy is the key, while VDOP is more relevant for airborne sources, such as for aircraft, where vertical accuracy is more desired. When both horizontal and vertical accuracy are needed, the metric of GDOP is preferred. In such cases, minimizing GDOP is thus a primary objective in various localization applications, beyond satellite navigation, including mobile sensor networks and ground-based systems, where GDOP analysis is extended to the time/angle of departure and arrival measurements [10,18,19,20,21].

Given the importance of minimizing GDOP in localization, various methods have been developed to determine optimal sensor placements. These methods include iterative solutions to optimization problems if extra sensors are added to the previous system [3]. Another method is the graph-based approach that combines regular polygons, tetrahedrons, and symmetric cones to achieve optimal topologies [11,22,23,24]. The locations of minimum GDOP are found at the tips of cones or the centers of polyhedrons, and we refer to these locations as designated min-GDOP points.

### 1.1. Fixed Versus Mobile Access Node Deployment

In many applications, sensors or access nodes are fixed after the optimal configuration topology is implemented. This fixed deployment offers the advantage of a one-time installation cost and eliminates mobility maintenance. However, such a setup lacks the adaptability to dynamic monitoring needs or changing environmental conditions. For instance, if a target moves out of the designated min-GDOP points, or new obstacles arise, the network may become suboptimal. To overcome these limitations, unmanned aerial vehicles (UAVs) have recently been applied in positioning applications due to their low cost, reusability, and high maneuverability [25]. In these geolocation applications with UAVs, the metric of GDOP is an important factor, serving as a design criterion or evaluation benchmark [26,27,28,29,30,31,32,33]. In [26], a distance-dependent noise model is introduced into the GDOP formulation to evaluate target tracking errors in UAV swarm systems. In [27], multiple UAVs are used to collect measurements among cooperative sensors, and a positioning algorithm is developed using the Riemannian manifold gradient. In this approach, the GDOP is incorporated to adapt the iterative descent rate of the manifold gradient, guiding the optimization toward the fastest descent direction. In [28], the authors use an Extended Kalman Filter to fuse Inertial Measurement Unit data and design UAV trajectories that minimize GDOP, in order to maintain accurate relative localization, particularly during GPS outages. In [29,30], a GDOP model is proposed for hybrid time difference of arrival (TDOA) and frequency difference of arrival (FDOA) positioning systems. The UAV swarm trajectory is optimized to achieve the best positioning geometry, thereby enhancing target position and velocity estimation accuracy. In [31], multi-source Direction of Arrival (DOA) localization using UAV cluster systems is investigated. The simulation results show that the localization accuracy is influenced by both DOA estimation performance and the resulting GDOP. Additionally, ref. [32] derives a GDOP expression for maritime multi-UAV passive positioning, while ref. [33] proposes a regional localization scheme using UAVs as access nodes to improve DOP (Dilution of Precision) and coverage.

### 1.2. A Trade-Off Between the Fixed and Mobile Access Node Deployment

While many recent positioning applications utilize multiple UAVs or UAV swarms [26,27,28,29,31,32,33], many fixed sensor placement strategies predate the widespread use of UAVs. This paper introduces a single UAV collaborating with existing fixed access nodes for positioning. This approach adds flexibility to fixed sensor placement while avoiding the cost of establishing completely new UAV-based configurations, representing a compromise between an all-fixed sensor placement and a solely UAV system. In this approach, the fixed sensor placement plays an important role, and an optimal placement, such as minimum GDOP placement, is preferred. An additional benefit of minimum GDOP is that it provides more potential to yield lower HDOP or VDOP for applications where horizontal or vertical positioning is the primary concern. Therefore, it makes sense to use the minimum GDOP placements as a foundation to begin with.

For a placement designed for minimum GDOP using a graph method, the minimum GDOP is only achieved at the designated min-GDOP points. If the unknown node is not located precisely at these points, the GDOP value increases, as the conditions for minimum GDOP are no longer satisfied. Recognizing that the unknown node is likely to be anywhere within a given area in practical scenarios, fixed sensor placements, once deployed, become inherently suboptimal. Introducing a mobile UAV offers a solution through its adaptability. The UAV can dynamically adjust its position to enhance GDOP and thus positioning accuracy. This inherent adaptability is a significant benefit of integrating UAVs with existing fixed sensor networks.

For a given number of access nodes *N*, multiple configuration topologies exist for minimum GDOP placement [11,17,24]. Among these topologies, we consider a particular placement in the category of symmetric cones, namely the zenith-horizon placement [17]. In this placement, one access node is placed along the z-axis, and the other nodes are positioned on a symmetric 3D cone. This topology is well suited to our strategy, as it comprises two parts: one with a node on the z-axis, and the other with nodes on a cone centered around the z-axis. We introduce a UAV-assisted placement by replacing the node on the z-axis with a UAV or a drone, which moves in circles centered on the z-axis. If the UAV moves in any way other than up and down along the z-axis, the original minimum GDOP topology of the fixed sensor placement is disrupted. We consider this disruption beneficial, because the minimum GDOP placement has only one designated min-GDOP point, located at the cone’s tip. In contrast, we need to evaluate the averaged GDOP across an area, rather than at a single point (the origin). Furthermore, we want to retain most of the advantages of the original minimum GDOP topology, and the zenith-horizon setup seems a promising fit for our proposed approach, allowing the other N−1 nodes to preserve the primary part of the structure.

With the UAV serving as an access node, its mobility provides flexible positions for taking measurements. Theoretically, measurements from the UAV can be taken anywhere along its flight path. We assume that one measurement is selectively taken from a particular UAV trajectory at a specific time instant. When the unknown node is located away from the designated min-GDOP points, evaluating location accuracy across the area at non-designated min-GDOP points becomes more relevant. Considering that the unknown node can be located anywhere within a given area, using the averaged HDOP and GDOP over the area provides a more appropriate metric than considering those values for a single unknown node position [34,35,36]. The goal of this paper is to examine UAV flight paths with different heights and angles and to find the best positions for taking measurements to achieve the lowest averaged HDOP or/and GDOP over the area. The complexity of this optimization makes obtaining a closed-form solution challenging. The contributions of this paper are summarized as follows:

- Numerical solutions are proposed to identify the best UAV position for measurement acquisition, producing the lowest averaged HDOP and/or GDOP over the given area.
- Extensive simulations are performed with the following key findings:
  - Achieving the minimum average HDOP and GDOP often requires distinct UAV positions, given the total number of access nodes and the size of the search area. This typically makes simultaneous minimization of both HDOP and GDOP with a single UAV flight path unattainable.
  - For applications prioritizing minimum average HDOP with a fewer number of access nodes, aligning the UAV’s XY-plane angle with the stationary nodes’ angles, offset by $60^{\circ }$, is advantageous. This angular alignment becomes less significant as the number of access nodes grows.
  - In applications where both HDOP and GDOP are important, appropriate UAV positions can be determined by considering acceptable trade-off levels.
  - The extent to which increasing the number of access nodes improves average HDOP and GDOP is quantified for a given area.

**Notation 1.**  *The transposes of vector a and matrix A are denoted by $a^{T}$ and $A^{T}$, respectively. I is an identity matrix with an appropriate size. Notation trace (A) denotes the trace of matrix A. ‖x‖ represents the norm of x. Vector 1 contains all 1 with an appropriate size.*

## 2. Concept of Geometric Dilution of Precision and Horizontal Dilution of Precision

The metric of GDOP mostly originates from geolocalization applications, where the position of an unknown node is estimated using the distance measurement from a number of access nodes. Suppose the unknown node is located at $p_{u}=\left(x_{u},y_{u},z_{u}\right)$, where $\left(x_{u},y_{u},z_{u}\right)$ represents the coordinate of $p_{u}$ in the 3D Cartesian coordinate system, and the *j*-th access node is located at $p_{j}=\left(x_{j},y_{j},z_{j}\right),j=1,\ldots N$, *N* is the number of access nodes, and N≥4 is assumed in this paper. The 3D distance measurement between the *j*-th access node and the unknown node is expressed as [1,37]

(1) $$\begin{array}{cc} r_{j} & =\;\|p_{j}-p_{u}\|+\varepsilon_{j} \end{array}$$

(2) $$\begin{array}{cc} = & \sqrt{{\left(x_{j}-x_{u}\right)}^{2}+{\left(y_{j}-y_{u}\right)}^{2}+{\left(z_{j}-z_{u}\right)}^{2}}+\varepsilon_{j}, \end{array}$$

where $\varepsilon_{j}$ is the Gaussian-distributed observation error with zero mean and identical variance $\sigma^{2}$ for all access nodes. Ignoring the noise, the distance measurement can be linearized by Taylor series expansion around an approximate position of ${\hat{p}}_{u}=\left({\hat{x}}_{u},\;{\hat{y}}_{u},\;{\hat{z}}_{u}\right)$. We have $\hat{b}=As$ [1,11,38,39], where vector $\hat{b}=\left(|\right|p_{1}-{\hat{p}}_{u}\left||,\ldots ,|\right|p_{N}-{\hat{p}}_{u}{\left||\right)}^{T}$, and $s={\left(x_{u}-{\hat{x}}_{u},\;y_{u}-{\hat{y}}_{u},\;z_{u}-{\hat{z}}_{u}\right)}^{T}$ is the vector for position error, and $A\in R^{N\times 3}$ is the geometric matrix [23,38,40]

(3) $$\begin{array}{cc} A & =[e_{x}\;\;e_{y}\;\;e_{z}] \end{array}$$

where

(4) $$\begin{array}{cc} e_{x} & ={\left(e_{x,1},\ldots ,e_{x,N}\right)}^{T},e_{x,j}=\frac{x_{j}-{\hat{x}}_{u}}{\left|\right|p_{j}-{\hat{p}}_{u}\left|\right|},j=1,\ldots ,N \end{array}$$

(5) $$\begin{array}{cc} e_{y} & ={\left(e_{y,1},\ldots ,e_{y,N}\right)}^{T},e_{y,j}=\frac{y_{j}-{\hat{y}}_{u}}{\left|\right|p_{j}-{\hat{p}}_{u}\left|\right|},j=1,\ldots ,N \end{array}$$

(6) $$\begin{array}{cc} e_{z} & ={\left(e_{z,1},\ldots ,e_{z,N}\right)}^{T},e_{z,j}=\frac{z_{j}-{\hat{z}}_{u}}{\left|\right|p_{j}-{\hat{p}}_{u}\left|\right|},j=1,\ldots ,N \end{array}$$

The geometry of the access nodes significantly affects the accuracy of the estimated state vector s. The least squares solution for s is $\tilde{s}={\left(A^{T}A\right)}^{-1}A^{T}\hat{b}$ [23,38]. Assuming white measurement noise with variance $\sigma^{2}$, the resulting covariance matrix is $\mathrm{Cov}\left(\tilde{s}\right)=\sigma^{2}{\left(A^{T}A\right)}^{-1}$. To quantify this geometric impact, the GDOP and HDOP metrics are used [1,17,34,39,41], and their definitions are given as follows:

(7) $$\begin{array}{cc} \mathrm{GDOP} & =\sqrt{\mathrm{trace}{\left(A^{T}A\right)}^{-1}}=\sqrt{q_{1}+q_{2}+q_{3}} \end{array}$$

(8) $$\begin{array}{cc} \mathrm{HDOP} & =\sqrt{q_{1}+q_{2}},\;\mathrm{VDOP}=\sqrt{q_{3}} \end{array}$$

where the diagonal elements of the matrix ${\left(A^{T}A\right)}^{-1}$ are collected in the vector $q={\left[q_{1}\;q_{2}\;q_{3}\right]}^{T}$. For applications where precise location in the XY-plane is most important, HDOP is the primary metric. Conversely, GDOP is used when accurate 3D positioning (in the XYZ-plane) is essential. This paper focuses on GDOP and HDOP, and also discusses VDOP.

The entries in matrix A are composed of ratios of distances (as seen in entries), making the resulting GDOP value unitless. The value depends solely on the geometry of the access nodes, which collect the measurements, relative to the unknown node. In other words, the values of GDOP, HDOP, or VDOP are not affected by the noise variance.

It is worth noting that the definition of GDOP in (7) assumes that the noise in each measurement is independent and has the same variance. However, this assumption may not hold in certain applications, particularly when combining different systems. In such cases, the weighted GDOP (WGDOP) is commonly used [37,39,42]:

(9) $$\begin{array}{c} {\mathrm{GDOP}}_{W}=\sqrt{\mathrm{trace}{\left(A^{H}WA\right)}^{-1}} \end{array}$$

where W is a weighting matrix, which is directly related to the inverse of the covariance matrix of measurement noise.

## 3. Optimal Placement for Minimum GDOP

Given the total number of access nodes *N*, the minimum GDOP value in (7) is $G_{\min}=3/\sqrt{N}$ [17,24]. The condition to achieve the minimum is [24]

(10) $$\begin{array}{cc} A^{T}A & =\frac{N}{3}I. \end{array}$$

For the sake of completeness, the proof for (10) is provided in Appendix A. Two primary categories of sensor placement have been traditionally identified for achieving optimal minimum GDOP. One approach uses combinations of regular polyhedra, specifically tetrahedra, hexahedra (cubes), and icosahedra [11,22,24]. In these setups, the minimum GDOP occurs at the centroid of the resulting 3D shapes, often requiring negative Z-coordinate placements that can be challenging to implement in practical applications, except for underground or underwater scenarios. The second category utilizes symmetric 3D nested-cone arrangements [11,24], where the minimum GDOP is located at the vertex of the cones.

**Figure 1:**
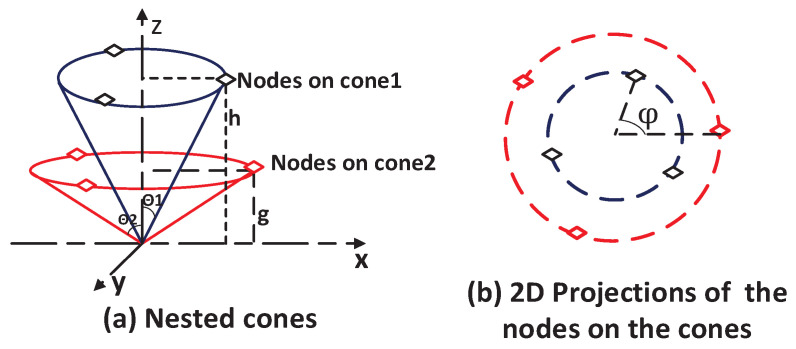
Spatial arrangement of six access nodes on nested 3D cones. (**a**) Three nodes are located on the outer cone defined by elevation *h* and angle $\theta_{1}$, while the other three are on the inner cone with elevation *g* and angle $\theta_{2}$. (**b**) Top-down (XY-plane) projection showing the relative positions of the access nodes.

**Figure 2:**
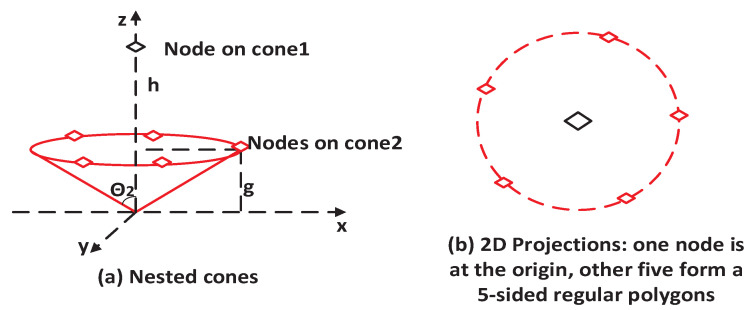
Configuration of six access nodes on nested 3D cones. (**a**) One access node is positioned on the *z*-axis at height *h* ($\theta_{1}=0$). The remaining five access nodes are located on a cone with elevation *g* and angle $\theta_{2}$. (**b**) XY-plane projection showing the 2D positions of the five access nodes.

The strategy of 3D symmetric nested cones can generate multiple placements for the minimum GDOP for a given number of access nodes. Take six access nodes as an example; Figure 1 and Figure 2 plot two of these optimal placements. Figure 1a plots two nested cones, where three nodes are placed on each cone, the first cone has a height *h* and opening angle $\theta_{1}$, and the second cone has a height *g* and opening angle $\theta_{2}$. To meet the minimum GDOP condition in (10), the angles $\theta_{1}$ and $\theta_{2}$ must satisfy the following relationship given in (11). Additionally, the access nodes on each cone, when projected onto the XY-plane, should form regular triangles [24].

(11) $$\begin{array}{c} n_{1}\cos^{2}\theta_{1}+n_{2}\cos^{2}\theta_{2}=\left(n_{1}+n_{2}\right)/3, \end{array}$$

where $n_{i},i=1,2$ is the number of nodes on the *i*-th cone. In Figure 1, $n_{1}=3=n_{2}$, whereas Figure 2 shows $n_{1}=1,n_{2}=5$. Figure 1b plots the projected 2D positions for the six access nodes marked by the diamonds with solid and dashed lines, respectively, and ψ is the separation angle between the two three-sided regular polygons.

Another optimal placement generated by the 3D symmetric nested cones for six access nodes, shown in Figure 2a, features a first cone with a special angle $\theta_{1}=0$. One access node is placed on this cone, along the z-axis. The other five nodes are placed on a second cone, with their projected 2D positions forming a five-sided regular polygon (Figure 2b). This configuration, also known as zenith-horizon placement, is used in satellite selection [17]. While $\theta_{2}$ is used in both Figure 1 and Figure 2, its specific value differs between the placements and must be solved individually to achieve the minimum GDOP.

It is worth noting that the proposed UAV-assisted placement configurations are more relevant to the topological structure depicted in Figure 2. Nevertheless, Figure 1 is also presented to provide a more complete description of the category of symmetric nested-cone placements used to achieve the minimum GDOP at the designated min-GDOP point, which is also the cone’s apex. To lay the groundwork for the UAV-assisted system design, it is beneficial to examine characteristics, such as the HDOP and GDOP, for a purely fixed node system.

## 4. HDOP for Zenith-Horizon Placement with All-Fixed Access Nodes

In this section, we examine the HDOP value for a minimum GDOP placement, the zenith-horizon placement, where all nodes are stationary. From (7) and (8), achieving low HDOP values is more likely when GDOP values are also low. Therefore, to maximize the potential for lower HDOP values, it is desirable to base the placement strategy on minimizing GDOP. It is important to remember that the minimum GDOP is realized solely when the unknown node is positioned at the cone’s tip, the designated min-GDOP point. Therefore, we will now examine the HDOP at this optimal location as well as at other non-designated points.

### HDOP for Designated Min-GDOP Point

We use the scenario with N=4 to illustrate the HDOP, where the unknown node is located at the designated min-GDOP point (the origin). The same procedure applies to a higher number of access nodes. A zenith-horizon configuration with six access nodes is illustrated in Figure 2. Based on the topology in the figure, the zenith-horizon configuration with four access nodes is composed of one node located on the z-axis with elevation *h* and three access nodes on a cone with opening angle $\theta_{2}$ and elevation *g*. The coordinates for the access node on the z-axis are

(12) $$\begin{array}{c} p_{0}=\left(x_{0},\;y_{0},\;h\right)=\left(0,\;0,\;h\right). \end{array}$$

The coordinates for the access nodes on the cone are given by

(13) $$\begin{array}{c} p_{i}=\left(x_{i},\;y_{i},\;z_{i}\right)=\left(\rho \sin\theta_{2}\cos\alpha_{i},\rho \sin\theta_{2}\sin\alpha_{i},g\right),i=1,2,3. \end{array}$$

where $\rho =g/\cos\theta_{2}$, and the 2D projections of the three nodes onto the XY-plane form a regular triangle, with angles defined by $\alpha_{i}=\frac{2\pi }{3}\left(i-1\right)$.

When the unknown node is positioned at the origin, which is the designated min-GDOP point, the geometric matrix (at the origin) $\bar{A}$ is given by (3)

(14) $$\begin{array}{c} \bar{A}=\left(\begin{array}{ccc} e_{x} & e_{y} & e_{z} \end{array}\right)=\left(\begin{array}{ccc} \frac{x_{0}}{h} & \frac{y_{0}}{h} & \frac{z_{0}}{h}\\ \frac{x_{1}}{\rho } & \frac{y_{1}}{\rho } & \frac{g}{\rho }\\ \frac{x_{2}}{\rho } & \frac{y_{2}}{\rho } & \frac{g}{\rho }\\ \frac{x_{3}}{\rho } & \frac{y_{3}}{\rho } & \frac{g}{\rho } \end{array}\right). \end{array}$$

Inserting the coordinates in (12) and (13) into (14), we obtain

(15) $$\begin{array}{ccc} \; & {\bar{A}}^{T}\bar{A}=\; & \;\;\;\;\;\;\left(\;\;\;\;\begin{array}{ccc} \sin^{2}\theta_{2}\sum_{i=1}^{3}\cos^{2}\alpha_{i} & 0 & 0\\ 0 & \sin^{2}\theta_{2}\sum_{i=1}^{3}\sin^{2}\alpha_{i} & 0\\ 0 & 0 & 1+3\cos^{2}\theta_{2} \end{array}\;\;\;\;\right).\;\; \end{array}$$

From (15), the GDOP and HDOP values are independent of the access nodes’ elevations due to the assumption that the unknown node is located at the origin. In this case, the value of HDOP is given by

(16) $$\begin{array}{c} {\mathrm{HDOP}}_{\mathrm{special}}=\frac{2}{\sqrt{3}\sin\theta_{2}}=\frac{2}{\sqrt{\left(N-1\right)}\sin\theta_{2}}, \end{array}$$

where the fact $\sum_{i=1}^{3}\cos^{2}\alpha_{i}=\sum_{i=1}^{3}\sin^{2}\alpha_{i}=3/2$ is used. From (16), the HDOP value decreases with a greater number of stationary nodes or as the angle $\theta_{2}$ approaches to $90^{\circ }$. Some practical systems often make it unrealistic to have $\theta_{2}$ close to $90^{\circ }$. For example, satellite systems require $\theta_{2}$ to be within the mask angle. The value of $\theta_{2}$ also impacts the value of VDOP, as described by ${\mathrm{VDOP}}_{\mathrm{special}}=\frac{1}{\sqrt{1+3\cos^{2}\theta_{2}}}$. This equation shows that VDOP becomes large as $\theta_{2}$ approaches $90^{\circ }$. Evidently, a trade-off exists between HDOP and VDOP.

Recognizing the relationship where a lower GDOP typically indicates the potential lower HDOP and VDOP values, it is therefore sensible to select $\theta_{2}$ to satisfy the minimum GDOP condition given in (10), where the diagonal elements of the matrix in (15) must be 4/3. The specific value of $\theta_{2}$ that fulfills this requirement can be determined by solving the resulting equations:

(17) $$\begin{array}{cc} \sin^{2}\theta_{2}\sum_{i=1}^{3}\cos^{2}\alpha_{i} & =4/3 \end{array}$$

(18) $$\begin{array}{cc} \sin^{2}\theta_{2}\sum_{i=1}^{3}\sin^{2}\alpha_{i} & =4/3 \end{array}$$

(19) $$\begin{array}{cc} 1+3\cos^{2}\theta_{2} & =4/3 \end{array}$$

Given the identities $\sum_{i=1}^{3}\cos^{2}\alpha_{i}=\sum_{i=1}^{3}\sin^{2}\alpha_{i}=3/2$, solving (19) is sufficient. The solution yields $\cos^{2}\theta_{2}=1/9$, which implies $\cos\theta_{2}=1/3$ and $\theta_{2}=70.53^{\circ }$. In fact, the same equality in (19) can also be obtained from (11), which states $n_{1}\cos^{2}\theta_{1}+n_{2}\cos^{2}\theta_{2}=\left(n_{1}+n_{2}\right)/3$, where $n_{1}=1,n_{2}=3,\theta_{1}=0$ in this specific scenario.

**Figure 3:**
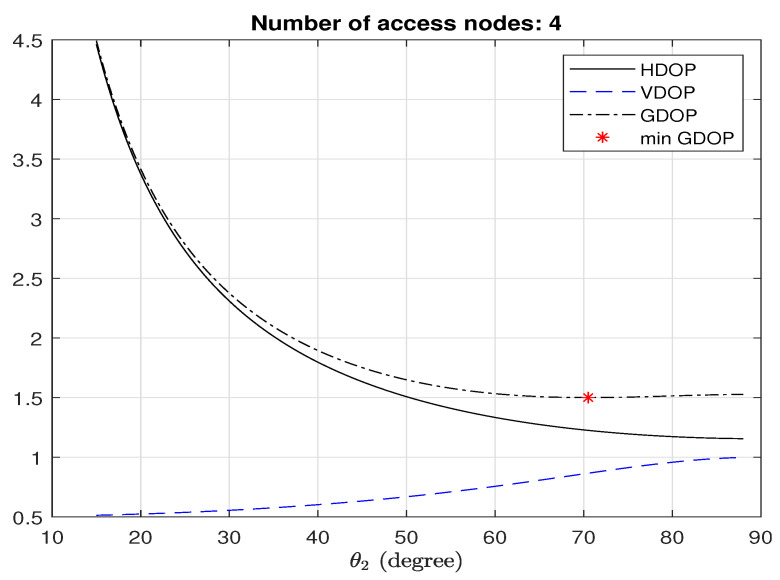
HDOP in (16), GDOP, and VDOP at the origin with respect to $\theta_{2}$.

Substituting $\sin\theta_{2}=\sqrt{8}/3$ into (16) gives an HDOP of $\sqrt{3/2}$. This value is consistent with that calculated using (8) and $q_{1}=q_{2}=q_{3}=\sqrt{3/N}$, which stems from the minimum GDOP condition in (10). Figure 3 plots the ${\mathrm{HDOP}}_{\mathrm{special}}$ in (16) against $\theta_{2}$. For comparison, the corresponding GDOP and VDOP are also plotted, where the * indicates the minimum GDOP point. Despite not yielding the absolute lowest HDOP at the minimum GDOP point, the reduced HDOP observed provides a valid justification for using the minimum GDOP placement strategy.

**Figure 4:**
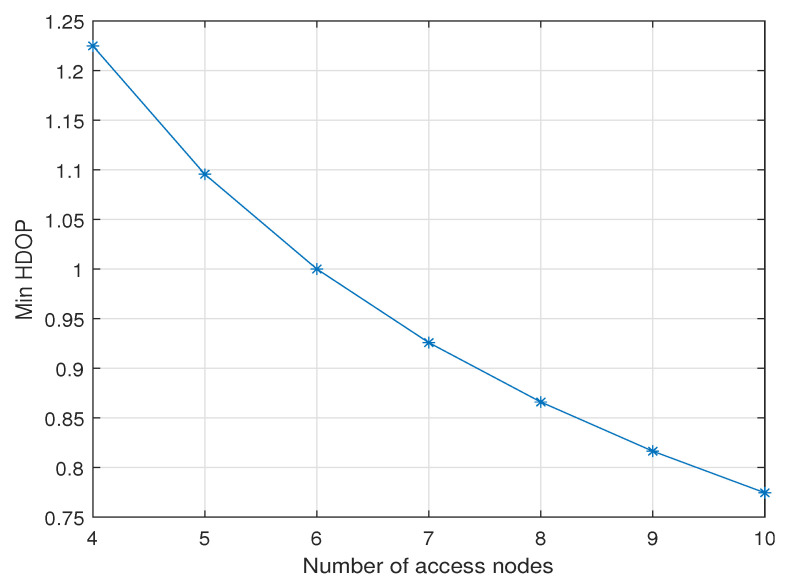
$H_{Gmin}$ at the origin with a different number of access nodes in zenith-horizon placement with fixed nodes.

An increase in the number of access nodes is generally expected to reduce HDOP. While the minimum GDOP value does not necessarily guarantee the absolute minimum HDOP, it remains valuable for examining the relationship between HDOP and the number of access points under the minimum GDOP condition specified in (10). Under this condition, we have ${\bar{A}}^{T}\bar{A}=\frac{N}{3}I$, leading to a minimum GDOP of $G_{min}=\frac{3}{\sqrt{N}}$. Consequently, the HDOP under this minimum GDOP condition is then $H_{Gmin}=\sqrt{\frac{3}{2}}G_{min}$. Figure 4 plots the value of $H_{Gmin}$ with different numbers of access nodes. The figure suggests a reduction of about 0.06∼0.12 in HDOP per additional access node, under the condition that the unknown node is located at the origin and with the optimal $\theta_{2}=70.53^{\circ }$ for minimum GDOP.

A key characteristic of a minimum GDOP placement is that the lowest GDOP is not broadly available but is confined to specific locations, identified as the designated min-GDOP points, and these are the tips of the cones in the zenith-horizon setup. If the unknown node is not situated at these specific points, the corresponding GDOP value increases because the conditions for minimum GDOP are no longer met. Consequently, the HDOP value increases and the benefits of the minimum GDOP placement cannot be realized. In practical scenarios, the unknown node’s location is variable within an area. Consequently, assessing location accuracy across this entire area, rather than solely at specific minimum GDOP points, offers a more practical evaluation. Moreover, using the average HDOP and GDOP over the service area provides a more representative performance metric than considering values at a single, arbitrary node position [34,35,36]. To better address this need for area-wide accuracy, we next introduce the integration of a UAV.

## 5. UAV-Assisted Placement

Consider a minimum GDOP topology with the zenith-horizon structure, depicted in Figure 2, comprising N access nodes. In this setup, N−1 nodes are fixed on a cone with an opening angle $\theta_{2}$, chosen to satisfy the minimum GDOP condition (10) for this placement. While the zenith-horizon structure achieves a minimum GDOP, the minimum is realized only when the unknown node is located at the origin. If the unknown node is located anywhere in an area, the advantage of this minimum GDOP vanishes.

As we focus our attention on the averaged HDOP and GDOP over an area, it is desirable to select a placement which already achieves the minimum GDOP, so that higher potentials can be attained for lower averaged HDOP and GDOP values. To introduce UAV flexibility into the fixed sensor network, we propose releasing one of the existing nodes and deploying a UAV in its place. The zenith-horizon configuration is particularly well suited for this hybrid deployment. We replace the node initially located on the z-axis by a UAV, while keeping the stationary nodes situated at the same cone. Our objective is to improve localization accuracy throughout the entire area while keeping the total number of measurements constant at *N*. This involves N−1 measurements from the fixed access nodes and one measurement from the UAV at a defined point along its path. It is important to note that the UAV measurement is not taken evenly at time intervals. Instead, it is captured when the UAV reaches a position that yields the lowest average HDOP or GDOP value, avoiding the non ideal positions where the average HDOP/GDOP values are relatively high.

Our proposed strategy aims to pinpoint an optimal UAV position for a measurement that minimizes the *averaged* HDOP and/or GDOP across the area of interest. This straightforward and easily deployable system offers a compelling balance between the inherent structure of fixed sensor networks and the dynamic adaptability provided by a UAV. By acknowledging the realistic scenario of an unknown node’s presence within a defined area, rather than a fixed point, our approach leverages the well-established GDOP metric, extending its utility from a single-point evaluation to a more relevant area-wide average for randomly located targets. Consequently, the proposed optimization strategy for determining the UAV trajectory that minimizes this averaged DOP presents a viable and robust path towards enhanced localization accuracy.

### 5.1. Geometric Matrix for UAV-Assisted Placement

**Figure 5:**
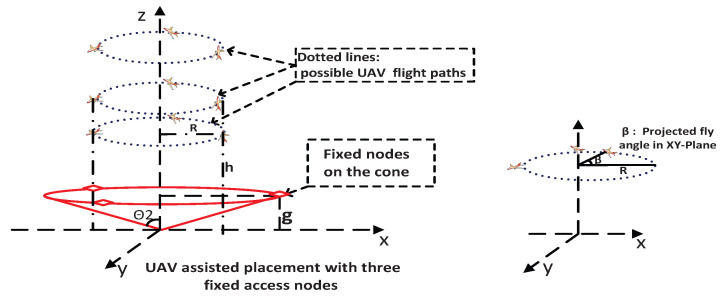
UAV-assisted placement with three stationary access nodes. (**Left**) The stationary access nodes and possible UAV flight paths in circles with different heights and radii. (**Right**) One UAV path in a circle.

As shown in Figure 5, when the UAV moves along its trajectory, its coordinates at the *n*-th position, denoted by $p_{0}\left(n\right)$, are expressed as follows:

(20) $$\begin{array}{c} p_{0}\left(n\right):\;\left(R\;\cos\beta (n),R(n)\;\sin\beta (n),h(n)\right),n=1,\;\ldots ,\;N_{p} \end{array}$$

where $\beta \left(n\right)\in \left(0,360^{\circ }\right),N_{p}$ is the total potential number of measurement positions at the UAV trajectory, R(n) is the horizontal distance of the UAV from the origin at the *n*-th position, and h(n) is the UAV’s elevation at the *n*-th position. For notation simplicity, we drop the index (n) in the rest of the paper.

Next, we examine the geometric matrix for the UAV-assisted system under two movement scenarios for the UAV: (1) movement solely along the vertical z-axis, which can be considered a special case of the cylindrical movement with radius R=0 as depicted in Figure 5; and (2) movement along the surface of a cylinder centered on the z-axis with R≠0 illustrated in Figure 5, where the dotted circles represent potential paths for the UAV, and β denotes the projected angle in the XY-plane.

For our proposed strategy, the specific flight path of the UAV is not critical, provided that the three-dimensional flight space is adequately divided and sampled for DOP calculation. A cylindrical flight path is chosen in this work for ease of UAV maneuverability, as sharp $90^{\circ }$ turns, such as those required in a square trajectory, are generally more challenging for UAVs to execute.

#### 5.1.1. UAV on Z-Axis

This movement scenario corresponds to R=0 in Figure 5. The coordinates of the UAV is $p_{0}=\left(0,0,h\right)$. For illustrative purposes, let us consider three stationary access nodes situated on the cone with elevation *g* and opening angle $\theta_{2}$. Their coordinates, denoted by $p_{i},i=1,2,3$, are specified in (13). The topological arrangement in this setup mirrors that in Figure 2; however, it features three nodes positioned on the cone, in contrast to the five illustrated in the figure.

For applications primarily concerned with localization in a 2D plane, one can assume, without loss of generality, that the height of the unknown node is zero. In this case, the coordinates of the unknown point, which is located anywhere in the area and is not necessarily at the origin, are given by $p_{p}=\left(x_{p},y_{p},0\right),x_{p},y_{p}\neq 0$. By inserting these coordinates into (3), the geometric matrix is obtained as shown below.

(21) $$\begin{array}{cc} A & =\left(\begin{array}{ccc} \frac{x_{0}-x_{p}}{r_{0}} & \frac{y_{0}-y_{p}}{r_{0}} & \frac{z_{0}}{r_{0}}\\ \frac{x_{1}-x_{p}}{r_{1}} & \frac{y_{1}-y_{p}}{r_{1}} & \frac{z_{1}}{r_{1}}\\ \frac{x_{2}-x_{p}}{r_{2}} & \frac{y_{2}-y_{p}}{r_{2}} & \frac{z_{2}}{r_{2}}\\ \frac{x_{3}-x_{p}}{r_{3}} & \frac{y_{3}-y_{p}}{r_{3}} & \frac{z_{3}}{r_{3}} \end{array}\right)=\left(\begin{array}{ccc} \frac{-x_{p}}{r_{0}} & \frac{-y_{p}}{r_{0}} & \frac{h}{r_{0}}\\ \frac{x_{1}-x_{p}}{r_{1}} & \frac{y_{1}-y_{p}}{r_{1}} & \frac{g}{r_{1}}\\ \frac{x_{2}-x_{p}}{r_{2}} & \frac{y_{2}-y_{p}}{r_{2}} & \frac{g}{r_{2}}\\ \frac{x_{3}-x_{p}}{r_{3}} & \frac{y_{3}-y_{p}}{r_{3}} & \frac{g}{r_{3}} \end{array}\right) \end{array}$$

where $r_{i}=\sqrt{{\left(x_{i}-x_{p}\right)}^{2}+{\left(y_{i}-y_{p}\right)}^{2}+z_{i}^{2}},i=0,1,2,3$, $z_{0}=h,z_{i}=g,i=1,2,3$. Inserting $p_{0}=\left(0,0,h\right)$, the matrix $A^{T}A$ is given by

(22) $$\begin{array}{cc} \; & A^{T}A=\left(\begin{array}{ccc} \|e_{x}{\|}^{2} & e_{x}^{T}e_{y} & e_{x}^{T}e_{z}\\ e_{x}^{T}e_{y} & \|e_{y}{\|}^{2} & e_{y}^{T}e_{z}\\ e_{x}^{T}e_{z} & e_{y}^{T}e_{z} & \|e_{z}{\|}^{2} \end{array}\right) \end{array}$$

(23) $$\begin{array}{cc} \; & \;\;=\;\left(\;\;\begin{array}{ccc} \sum_{i=0}^{3}\;\frac{x_{p}^{2}}{r_{i}^{2}} & \sum_{i=0}^{3}\;\frac{x_{p}y_{p}}{r_{i}^{2}} & -\;\sum_{i=0}^{3}\;\frac{x_{p}z_{i}}{r_{i}^{2}}\\ \sum_{i=0}^{3}\;\frac{x_{p}y_{p}}{r_{i}^{2}} & \sum_{i=0}^{3}\;\frac{y_{p}^{2}}{r_{i}^{2}} & -\;\sum_{i=0}^{3}\;\frac{y_{p}z_{i}}{r_{i}^{2}}\\ -\;\sum_{i=0}^{3}\;\frac{x_{p}z_{i}}{r_{i}^{2}} & -\;\sum_{i=0}^{3}\;\frac{y_{p}z_{i}}{r_{i}^{2}} & \sum_{i=0}^{3}\;\frac{z_{p}^{2}}{r_{i}^{2}} \end{array}\;\;\right). \end{array}$$

Since $p_{p}$ is not the designated min-GDOP point, the condition in (10) for minimum ${\mathrm{GDOP}}_{d}$ is not satisfied. Therefore, $A^{T}A$ in (23) is no longer a scaled identity matrix.

While the HDOP values in Figure 4 are independent of the access node elevations, this is not the case for either: (i) HDOP evaluated at a non-designated min-GDOP point (Equation (23) is such a example), or (ii) when the access node $p_{0}$, i.e., the UAV, moves on a cylinder with R≠0 instead of solely along the z-axis, which we will discuss next.

#### 5.1.2. UAV on Cylinder with R≠0

In this case, the coordinates of the UAV are given by $p_{0}=\left(R\cos\beta ,R\sin\beta ,h\right),$ $R\neq 0,\beta \in (0,360^{\circ })$. The matrix $A^{T}A$ is given as shown below.

(24) $$\begin{array}{cc} A^{T}A\; & =\left(\begin{array}{ccc} \sum_{i=0}^{3}\;\frac{{\left(x_{i}-x_{p}\right)}^{2}}{r_{i}^{2}} & \sum_{i=0}^{3}\;\frac{\left(x_{i}-x_{p}\right)\left(y_{i}-y_{p}\right)}{r_{i}^{2}} & \;\sum_{i=0}^{3}\;\frac{\left(x_{i}-x_{p}\right)z_{i}}{r_{i}^{2}}\\ \sum_{i=0}^{3}\;\frac{\left(x_{i}-x_{p}\right)\left(y_{i}-y_{p}\right)}{r_{i}^{2}} & \sum_{i=0}^{3}\;\frac{{\left(y_{i}-y_{p}\right)}^{2}}{r_{i}^{2}} & \;\sum_{i=0}^{3}\;\frac{\left(y_{i}-y_{p}\right)z_{i}}{r_{i}^{2}}\\ \;\sum_{i=0}^{3}\;\frac{\left(x_{i}-x_{p}\right)z_{i}}{r_{i}^{2}} & \;\sum_{i=0}^{3}\;\frac{\left(y_{i}-y_{p}\right)z_{i}}{r_{i}^{2}} & \sum_{i=0}^{3}\;\frac{z_{p}^{2}}{r_{i}^{2}} \end{array}\right) \end{array}$$

### 5.2. Optimal UAV Position for Minimum Averaged HDOP and/or GDOP

If the unknown node is not situated at the origin (the designed min-GDOP point), and instead occupies other locations within the area, the HDOP value will generally vary depending on its specific location, as shown in Equations (23) and (24). Assuming a uniform probability distribution for the unknown node’s location ($p_{p}$) within a defined area, the area-averaged HDOP serves as a significant indicator of the localization error [34,35]. Our goal is to identify the best positions for the UAV to take measurements along its path to minimize the averaged HDOP and/or GDOP over the entire area. However, obtaining a closed-form solution to this problem is very challenging. This is because the HDOP and/or GDOP involves $\mathrm{trace}{\left(A^{T}A\right)}^{-1}$ (as shown in (23) and (24)), which is a non-convex function of the UAV position. Furthermore, the averaged DOPs are also dependent on the area’s size and shape.

To address the proposed optimization problem, we investigate a numerical solution. Given *N* access nodes, we first determine the opening angle $\theta_{2}$ of the cone where (N−1) stationary nodes are positioned, leveraging the minimum GDOP condition outlined in (11). Subsequently, the UAV’s movement is defined along a cylindrical path characterized by radius *R*, height *h*, and the XY-plane angle β ranging from $0^{\circ }$ to $360^{\circ }$. It is important to note that the UAV trajectory detailed in Section 5.1.1 represents a specific instance of this cylindrical path with the radius R=0. To simplify the problem, we fix the elevation of the stationary access nodes *g*. Now, the averaged HDOP and GDOP over the area are related to three parameters, (R,h, and β).

Without loss of generality, we consider a square area centered at the origin. However, it is important to note that the proposed numerical approach is not dependent on the shape or location of the area, provided that the region is properly partitioned and sampled for DOP computations over the area. The origin-centered area is chosen here because the zenith-horizon structure is also centered at the origin, which is intuitively expected to yield optimal estimation performance for a symmetric region. If the unknown node lies in an area which is not centered at the origin in practical scenarios, the zenith-horizon configuration may not produce the best results.

It is prudent to choose an appropriately sized area to avoid excessively high averaged HDOP and GDOP values, as arbitrarily large areas are impractical in real-world applications due to the associated unreliable estimates. Simulation trials are commonly employed to determine suitable area sizes that yield acceptable HDOP values for the proposed scheme. For a configuration with three stationary access nodes, a reasonable area size can be defined by the span of these nodes, calculated as $2g\tan\theta_{2}$. It is known that the GDOP values are smaller with more access nodes [2,43]. Similarly, this expectation holds for HDOP as well. For four and five stationary nodes, bigger area sizes can be chosen than the span of the stationary nodes.

#### 5.2.1. Proposed Numerical Solution

The following steps are implemented for the numerical solution to identify the optimal UAV trajectory:

- Given a set of values of (R,h, and β), calculate the averaged HDOP $\bar{H}\left(R,h,\beta \right)$ and GDOP $\bar{G}\left(h,R,\beta \right)$ over an area *S*. The average is performed across an area where the unknown node has an equal probability of being at any point inside. The area is discretized into a grid with $M_{x}$ and $M_{y}$ divisions along the X and Y coordinates, respectively. The averaged HDOP is then calculated by adding the HDOP values at all grids and dividing by the total number of grids.
  (25) $$\begin{array}{c} \bar{H}\left(R,h,\beta \right)=\frac{1}{M_{x}M_{y}}\sum_{i}^{M_{x}}\sum_{j=1}^{M_{y}}{\mathrm{HDOP}}_{i,j}\left(R,h,\beta \right), \end{array}$$
  where ${\mathrm{HDOP}}_{i,j}\left(R,h,\beta \right)$ represents the HDOP value calculated at the (i,j)-th grid point for the given R,h, and β. The averaged GDOP $\bar{G}\left(h,R,\beta \right)$ can be obtained in a similar manner.
  (26) $$\begin{array}{c} \bar{G}\left(R,h,\beta \right)=\frac{1}{M_{x}M_{y}}\sum_{i}^{M_{x}}\sum_{j=1}^{M_{y}}{\mathrm{GDOP}}_{i,j}\left(R,h,\beta \right), \end{array}$$
- Obtain the minimum averaged HDOP $\bar{H}\left(R,h,\beta \right)$ and GDOP $\bar{G}\left(h,R,\beta \right)$ over the parameters R,h, and β, and the corresponding parameters to achieve the minimum.
  (27) $$\begin{array}{cc} H_{m} & =min_{R,h,\beta }\bar{H}\left(R,h,\beta \right) \end{array}$$
  (28) $$\begin{array}{cc} G_{m} & =min_{R,h,\beta }\bar{G}\left(R,h,\beta \right). \end{array}$$
  The corresponding parameters to achieve the minimum are given by
  (29) $$\begin{array}{cc} \left(R_{oH},h_{oH},\beta_{oH}\right) & =\{\left(R,h,\beta \right)|\bar{H}\left(R,h,\beta \right)=H_{m}\} \end{array}$$
  (30) $$\begin{array}{cc} \left(R_{oG},h_{oG},\beta_{oG}\right) & =\{\left(R,h,\beta \right)|\bar{G}\left(R,h,\beta \right)=G_{m}\} \end{array}$$
  Visualizing the behavior of the averaged HDOP $\bar{H}\left(R,h,\beta \right)$ and GDOP $\bar{G}\left(h,R,\beta \right)$ as functions of three parameters R,h, and β necessitates a four-dimensional representation, which is challenging for intuitive analysis in simulations. To address this, we employ a two-step procedure which plots graphs with reduced dimensionality, in order to provide a clearer and more intuitive understanding of the relationships between the averaged HDOP $\bar{H}\left(R,h,\beta \right)$ and GDOP $\bar{G}\left(h,R,\beta \right)$ and their dependence on the parameters R,h, and β. Next, we detail the procedure for the averaged HDOP. A parallel procedure is then employed for the averaged GDOP.
  - Step 1. Find $H_{m}$ and $\left(R_{oH},h_{oH}\right)$ using the conditional minimum with respect to the angle β: For given values of *R* and *h*, we first find the minimum of the averaged HDOP by searching the full range of β from 0 to $360^{\circ }$. This conditional minimum value is then plotted in a 2D graph as a function of *R* and *h*. From this graph, we locate the minimum HDOP value, $H_{m}$, and identify the corresponding parameter values $\left(R_{oH},h_{oH}\right)$ that yield this minimum.
  - Step 2. Find $\beta_{oH}$: Using the determined value of $(R_{oH}$ (or $h_{oH}$), plot the 2D graph with respect to the range of *h* and β. The value of $\beta_{oH}$ is then obtained by locating the coordinates for value $H_{m}$.
- Depending on the applications, if the metric of HDOP is preferred (with no need to consider GDOP), then the UAV’s measurement position is chosen based on the tolerance threshold $H_{\varepsilon }=\left(1+\xi_{H}\right)H_{m}$, where $\xi_{H}\geq 0$ is a small number to control the threshold. When $\xi_{H}=0$, the threshold is equal to the minimum averaged HDOP, $H_{\varepsilon }=H_{m}$. The set of acceptable UAV positions $\left(\tilde{R},\tilde{h},\tilde{\beta }\right)$ is then defined as follows:
  (31) $$\begin{array}{cc} \left(\tilde{R},\tilde{h},\tilde{\beta }\right)=\left\{\left(R,h,\beta \right)\right|\bar{H}\left(R,h,\beta \right) & <H_{\varepsilon }\} \end{array}$$
  This set includes all combinations of radius *R*, height *h*, and angle β, for which the averaged HDOP, $\bar{H}\left(R,h,\beta \right)$ is less than the specified tolerance threshold $H_{\varepsilon }$.
- If the criterion of GDOP is desired (with no need to consider HDOP), then the UAV’s measurement position is determined for a tolerance threshold $G_{\varepsilon }=\left(1+\xi_{G}\right)G_{m}$, where $\xi_{G}\geq 0$. If $\xi_{G}=0$, the threshold equals the minimum averaged GDOP, $G_{\varepsilon }=G_{m}$. The set of acceptable UAV positions $\left(\hat{R},\hat{h},\hat{\beta }\right)$ is then defined as follows:
  (32) $$\begin{array}{cc} \left(\hat{R},\hat{h},\hat{\beta }\right)=\left\{\left(R,h,\beta \right)\right|\bar{G}\left(R,h,\beta \right) & <G_{\varepsilon }\} \end{array}$$
  This set includes all combinations of radius *R*, height *h*, and angle β, for which the averaged GDOP, $\bar{G}\left(R,h,\beta \right)$ is less than the specified tolerance threshold $G_{\varepsilon }$.
- When both HDOP and GDOP need to be considered, the UAV’s measurement position is chosen based on satisfying both tolerance thresholds $H_{\varepsilon }$ and $G_{\varepsilon }$ simultaneously:
  (33) $$\begin{array}{cc} \left(\overset{˘}{R},\overset{˘}{h},\overset{˘}{\beta }\right) & =\{\left(R,h,\beta \right)|\left[\bar{H}\left(R,h,\beta \right)<H_{\varepsilon }\right]\\ \; & \cap \left[\bar{G}\left(R,h,\beta \right)<G_{\varepsilon }\right]\} \end{array}$$
  The feasibility of finding a solution for (33) depends on the trade-off between the tolerances for the averaged HDOP and GDOP. If these tolerance thresholds are set too restrictively (i.e., too small), there might be no overlap between the set of positions satisfying $\left[\bar{H}\left(R,h,\beta \right)<H_{\varepsilon }\right]$ and that satisfying $\left[\bar{G}\left(R,h,\beta \right)<G_{\varepsilon }\right]$.

#### 5.2.2. Discussion on Computational Cost and Scalability

The proposed approach adds some degree of flexibility to fixed sensor placement by utilizing a single UAV. It avoids the cost of establishing completely new UAV-based configurations and offers a compromise between an all-fixed sensor placement and a solely UAV system. Our approach is based on the zenith-horizon structure for minimum GDOP, where the node on the z-axis is replaced by a UAV. The scalability of the approach is largely determined by the size of the existing zenith-horizon placement. Once the placement is available, given the number of access nodes, we only need to run the algorithm once to identify the optimal positions of the UAV. Therefore, the computational cost is incurred only once.

Since the proposed algorithm needs to compute the averaged DOP over the area where the unknown node is located for each UAV trajectory, the computational cost mainly depends on two factors: the size of the area and the volume of the 3D space where the UAV flies. The number of access nodes *N* contributes to the multiplication of the matrix $A^{T}A$, which is a 3×3 square matrix regardless of *N*. The inverse of a 3×3 matrix is quite manageable. Consequently, for a defined area and a specific number of access nodes (N), the computational cost is a one-time expense.

While the proposed algorithm is illustrated using a specific number of stationary access nodes with the zenith-horizon placement, it inherently offers flexibility regarding the number and placement of fixed sensor nodes. It can be implemented even when the fixed sensor node placements are not optimized for minimum GDOP, and the optimal UAV trajectory can still be identified as described previously. Nevertheless, the potential for improving the averaged DOP in such systems may be limited compared to the zenith-horizon setup, which is specifically structured to minimize GDOP.

## 6. Simulation Results and Discussions

In the simulations, we consider scenarios with a UAV integrated with three to six stationary access nodes (N=4,5,6,7). The stationary nodes are located on a cone with a fixed height of g=4. While this specific height serves as an example, the proposed algorithm’s implementation is not limited by this choice, and other heights are equally applicable. The value of angle $\theta_{2}$, which defines their placement, is determined based on the minimum GDOP condition in (10) for a zenith-horizon configuration and remains constant for a given number of access nodes *N*.

The UAV’s flight path consists of circular trajectories at varying radii (*R*) and heights (*h*) on a cylinder, with its position parameterized by the XY-plane angle β. At a desired measurement instance during its flight, the UAV collects measurements. This measurement, combined with those from the N−1 stationary access nodes, are used to compute the instantaneous HDOP and GDOP at a grip point in an area, where the unknown node can be randomly located.

The area under consideration is spatially discretized into a grid with a step size of 0.5 units along both the X and Y axes. Subsequently, the averaged HDOP and GDOP values are calculated using Equations (25) and (26). The discretization step size directly influences the computational cost of the algorithm; smaller step sizes lead to increased computations. However, since it is the minimum averaged DOP, rather than the individual DOP at each grid point, that determines the optimal UAV positions, a degree of tolerance is expected for the step size depending on the area size. This tolerance allows for a balance between the accuracy of the optimal UAV positions and the computational cost.

The simulation results (including the 2D graphs) are generated through the two-step procedure outlined in Section 5.2.

Given the application-specific criteria (31) and (32), the corresponding desired $\left(\hat{R},\hat{h},\hat{\beta }\right)$ and $\left(\overset{˘}{R},\overset{˘}{h},\overset{˘}{\beta }\right)$ are determined. For the combined case described in (33), the desired values $\overset{˘}{R}$, $\overset{˘}{h}$, and $\overset{˘}{\beta }$ are obtained by balancing the trade-off between the tolerance limits for averaged HDOP and GDOP. Given the extensive nature of our simulations and the resulting number of figures, the key observations are summarized below:

- For a given number of stationary nodes (N−1) and a defined search area for the unknown node, the optimal UAV position for minimizing average HDOP ($R_{oH},h_{oH},\beta_{oH}$) typically differs from the optimal position for minimizing average GDOP ($R_{oG},h_{oG},\beta_{oG}$). This suggests that a single UAV trajectory cannot simultaneously achieve both minimums.
- When minimizing average HDOP is the primary objective, we observed the following: For a smaller number of access nodes (e.g., three), there is a structured relationship between the fixed sensor arrangement and the ideal UAV flight angle β. Multiple UAV positions (specifically three in this case), corresponding to the XY-angles of the stationary nodes with a $60^{\circ }$ shift, can yield the minimum average HDOP. However, for a larger number of access nodes, the choice of β appears to have minimal impact on achieving this minimum.
- For applications requiring consideration of both HDOP and GDOP, a suitable UAV position can be determined by evaluating the trade-off based on acceptable tolerance thresholds for each metric.
- Increasing the number of stationary nodes generally leads to lower average HDOP and GDOP values.

### 6.1. UAV with Three Stationary Access Nodes

**Figure 6:**
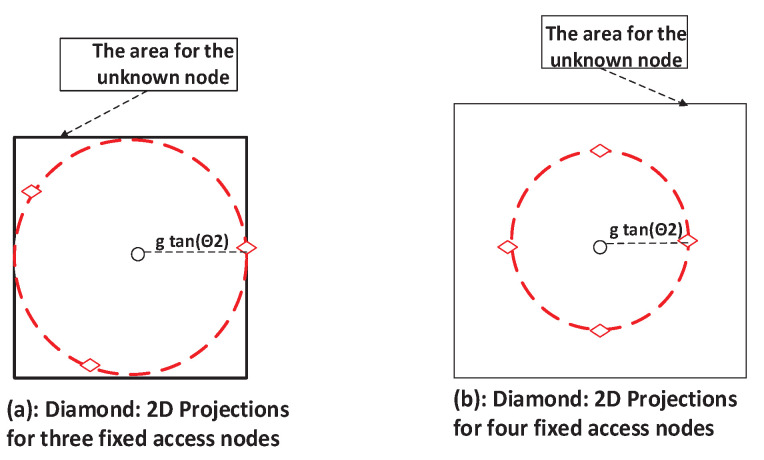
Area for unknown node with three stationary access nodes.

In this specific case with N=4 access nodes, the angle $\theta_{2}$ for minimum GDOP in a zenith-horizon configuration is $70.5^{\circ }$. Let $S_{s}$ be the area covered by the 2D projections of these stationary nodes, forming a square with side length $2g\tan\theta_{2}$. Assuming g=4, this yields a side length of approximately $2\times 4\times \tan(70.5^{\circ })\approx 22$ and thus an area of roughly 22×22. The target area *S*, used for averaging HDOP and GDOP, can be the same size as $S_{s}$ or larger, as depicted in Figure 6. For this analysis, we set the working area dimensions to be equal to those of $S_{s}$. This choice was guided by simulations aimed at maintaining acceptable levels of averaged HDOP and GDOP. Excessively high values of these metrics indicate substantial localization errors. In such cases, it may be beyond the UAV’s capability to reduce the DOPs to acceptable levels, especially considering that the stationary nodes are fixed and constitute the majority of the total access nodes in the system (N−1 compared to 1).

#### 6.1.1. Conditional Minimum for the Averaged HDOP and GDOP Given *R* and *h*

**Figure 7:**
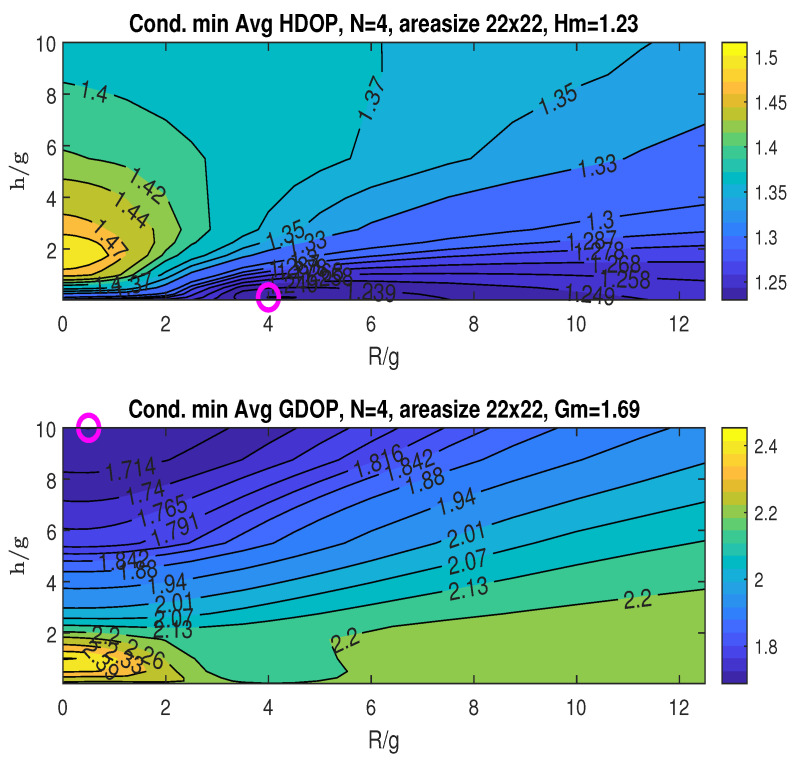
Conditional minimum averaged HDOP and GDOP over area (22×22) with three stationary nodes.

The upper graph in Figure 7 illustrates the conditional minimum averaged HDOP, obtained by minimizing over the angle β for specific values of *R* and *h*, following the two-step procedure detailed in Section 5.2.1. With *g* fixed at 4, we plot the HDOP contours against the normalized ratios R/g and h/g. These ratios provide a relative measure of the UAV’s flight radius *R* and height *h* with respect to *g*. The practical range for these ratios can be limited by the UAV’s physical constraints (power, weight, etc.) or by the value where further increases in R/g and h/g yield minimal improvement in the averaged minimum DOP. In our simulations, we rely on the latter criterion when selecting the ranges for R/g and h/g. The simulations exclude the special case where h/g=0 (i.e., h=0), because this condition would eliminate the UAV, which contradicts the fundamental structure of the proposed scheme. Furthermore, this condition tends to result in a rank-deficient geometric matrix at the origin, leading to ill-posed localization problems.

Finding the minimum averaged HDOP across the plotted ranges of R/g and h/g, as per the first step of the two-step procedure, we obtain $H_{m}=1.23$, as indicated in the plot title. This minimum occurs at $\left(R_{oH}/g,h_{oH}/g\right)=\left(4,0.125\right)$, marked by an “o” in the plot.

The bottom graph in Figure 7 shows the conditional minimum averaged GDOP plotted against the ratios R/g and h/g. The minimum GDOP across the considered ranges of R/g and h/g is $G_{m}=1.69$, which is also listed in the figure’s title. The minimum is attained at $\left(R_{oG}/g,h_{oG}/g\right)=\left(0.5,10\right)$, as indicated by the “o” marker in the plot. The graphs in Figure 7 demonstrate that the minimum HDOP ($H_{m}$) and minimum GDOP ($G_{m}$) occur at different *R* and *h* values.

#### 6.1.2. Averaged HDOP and GDOP with Respect to *h* and β for a Fixed *R*

In this simulation, we analyze the averaged HDOP and GDOP as functions of *h* and β, while keeping *R* constant at the value with $R_{oH}/g=4$ or $R_{oG}/g=0.5$.

**Figure 8:**
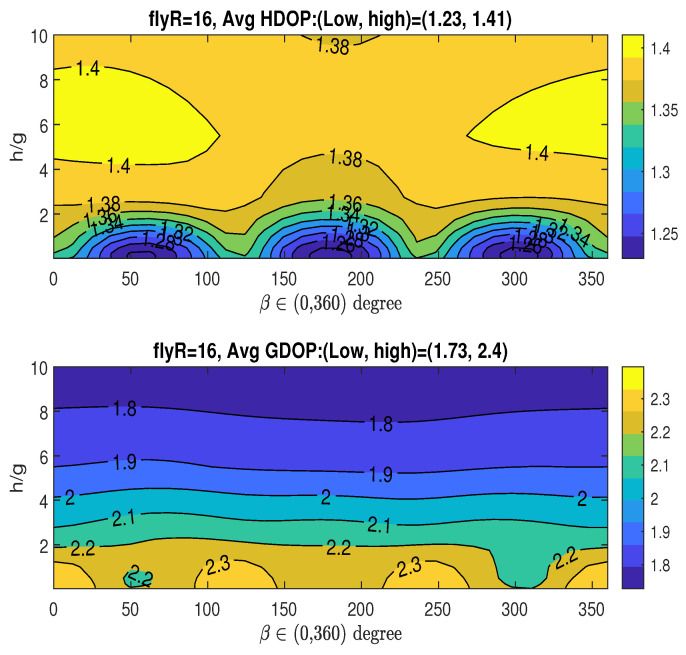
Averaged HDOP and GDOP over area (22×22) with three stationary nodes.

**Figure 9:**
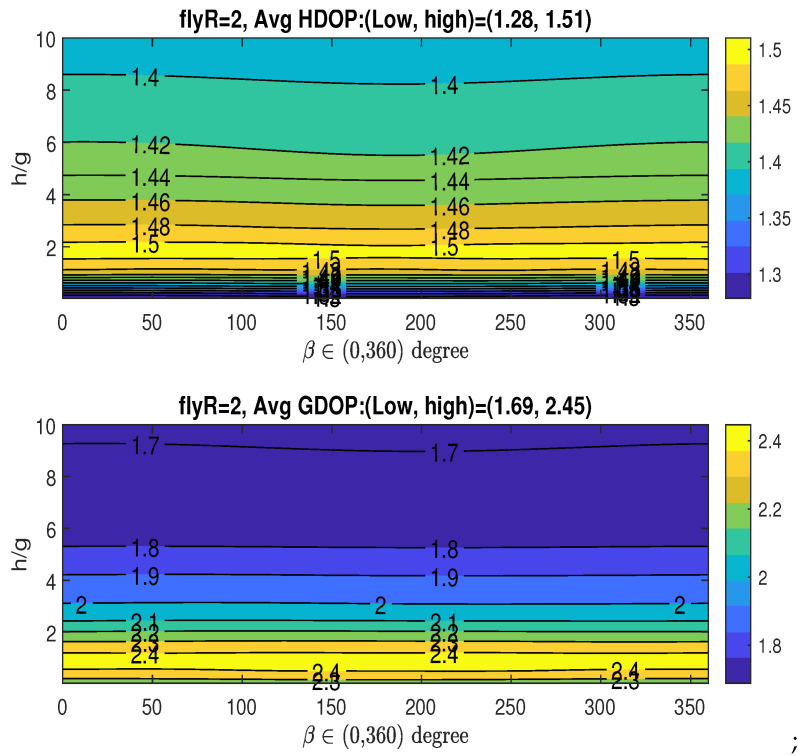
Averaged HDOP and GDOP over area (22×22) with three stationary nodes.

- Case 1: $R_{oH}/g=4$
  Given that $R_{oH}/g=4$, i.e., R=16, which achieves the minimum averaged HDOP ($H_{m}$), the upper graph in Figure 8 shows how the averaged HDOP varies with the angle $\beta \in (0,360^{\circ })$ and the ratio h/g. The HDOP values range from 1.23 to 1.41, as stated in the title. The figure reveals the existence of multiple optimal angles for β, which fall within the approximate ranges: $\beta_{oH}\in \left(50^{\circ }∼60^{\circ }\right),\left(172^{\circ }∼186^{\circ }\right),\left(298^{\circ }∼310^{\circ }\right)$. Considering the midpoints of the identified optimal β ranges, and recalling the XY-projected angles of the three fixed access nodes given by $\alpha_{i}=\frac{2\pi }{3}\left(i-1\right)$ for i=0,1,2 (specifically, $0^{\circ },120^{\circ }$, and $240^{\circ }$), an interesting observation arises: the optimal angles $\beta_{oH}$ are in close proximity to $\alpha_{i}+60^{\circ },i=0,1,2$. Acquiring a measurement from the UAV along its trajectory at any of these three potential β values, when combined with the parameters $\left(R_{oH}/g,h_{oH}/g\right)$, results in the lowest average HDOP.
  Based on the figure, we have the following observations regarding the averaged HDOP: achieving its minimum value allows for multiple optimal angular positions ($\beta_{oH}$) for UAV data acquisition, specifically within the approximate ranges of $\left(50^{\circ }∼60^{\circ }\right),\left(172^{\circ }∼186^{\circ }\right),$ and $\left(298^{\circ }∼310^{\circ }\right)$. Notably, these optimal UAV angles show a geometric relationship with the angular placement of the three stationary access nodes ($\alpha_{i}=0^{\circ },120^{\circ },240^{\circ }$), as the $\beta_{oH}$ ranges are centered around $\alpha_{i}+60^{\circ }$. This suggests a structured interaction between the fixed sensor arrangement and the ideal UAV measurement locations for minimizing horizontal positioning error. Therefore, acquiring measurements at these specific $\beta_{oH}$ ranges, in conjunction with the determined optimal radius and height $\left(R_{oH}/g,h_{oH}/g\right)$, is crucial for attaining the lowest average HDOP in this particular setup.
  Given $R_{oH}/g=4$, the bottom graph in Figure 8 illustrates the averaged GDOP as a function of the angle $\beta \in (0,360^{\circ })$ and the ratio h/g. The GDOP values span from 1.73 to 2.4, as indicated in the title. The previously determined minimum averaged GDOP of $G_{m}=1.69$ in the bottom graph in Figure 7 is not achievable, since $R_{oH}/g=4$ was optimized for $H_{m}$ and not necessarily for $G_{m}$. Consequently, when the UAV’s parameters $\left(R_{oH}/g,h_{oH}/g,\beta_{oH}\right)$ are chosen to minimize HDOP ($H_{m}$), the resulting averaged GDOP unfortunately reaches a relatively high value, around 2.2, as shown in the figure.
  If an application requires consideration of both HDOP and GDOP, and the UAV keeps the same flight radii with $R_{oH}/g=4$, a compromise can be achieved by selecting appropriate values for *h* and β and allowing the averaged HDOP and GDOP to deviate slightly from their absolute minimum values. For instance, one could select tolerance parameters $\xi_{H}=0.122$ and $\xi_{G}=0.066$, resulting in tolerance thresholds of $H_{\varepsilon }=1.38$ and $G_{\varepsilon }=1.8$, respectively, as defined in (33). This trade-off can be realized by selecting $\overset{˘}{h}/g$ close to 10 and setting the angle $\overset{˘}{\beta }=180^{\circ }$, which corresponds to $\alpha_{1}+60^{\circ }=120^{\circ }+60^{\circ }$, while maintaining $\overset{˘}{R}=R_{oH}$. For this specific scenario, it is important to note that only one optimal range for angle $\overset{˘}{\beta }$ is identified (associated with $\alpha_{1}$), which contrasts with situation where the sole objective is minimizing the averaged HDOP, which can yield three optimal ranges for β (linked to $\alpha_{0},\alpha_{1}$, or $\alpha_{2}$).
- Case 2: $R_{oG}/g=0.5$
  In this case, we examine the averaged HDOP and GDOP for $R_{oG}/g=0.5$ (i.e., R=2), which results in the minimum averaged GDOP, $G_{m}=1.69$. Figure 9 illustrates the averaged HDOP and GDOP as a function of h/g and β. The averaged HDOP values are within the range of (1.28∼1.51), while the GDOP values are within the range of (1.69∼2.45). From the lower graph, the value of $G_{m}$ is achieved for h/g>9.5 across the entire range of $\beta \in (0^{\circ }∼360^{\circ })$.
  Since we are using $R_{oG}$ (instead of $R_{oH}$), the minimum HDOP ($H_{m}=1.23$) shown in the upper graph of Figure 8 is not attained here. For applications where both HDOP and GDOP are important, a trade-off can be found by choosing $\overset{˘}{h}/g$ between 9 and 10, and any angle $\overset{˘}{\beta }$ from $0^{\circ }$ to $360^{\circ }$, while keeping $\overset{˘}{R}=R_{oG}$. With these selections, the averaged GDOP is roughly 1.7, and the averaged HDOP is about 1.4. Remarkably, the specific value of β has minimal impact on the resulting averaged GDOP and HDOP, which remain approximately 1.7 and 1.4, respectively.

### 6.2. UAV and Four Stationary Access Nodes

With a total of N=5 access nodes in this scenario, the minimum GDOP condition for zenith-horizon placement necessitates an angle of $\theta_{2}=65.9^{\circ }$. The spatial span of the four fixed access nodes is $2g\tan(\theta_{2})=17.9$. Given the addition of one more node compared to the scenario in Section 6.1, we now investigate how the size of the unknown node’s localization area affects the results of our proposed optimization method. To achieve this, two different area sizes (30×30) and (40×40) are considered.

#### 6.2.1. Area Size (30×30)

**Figure 10:**
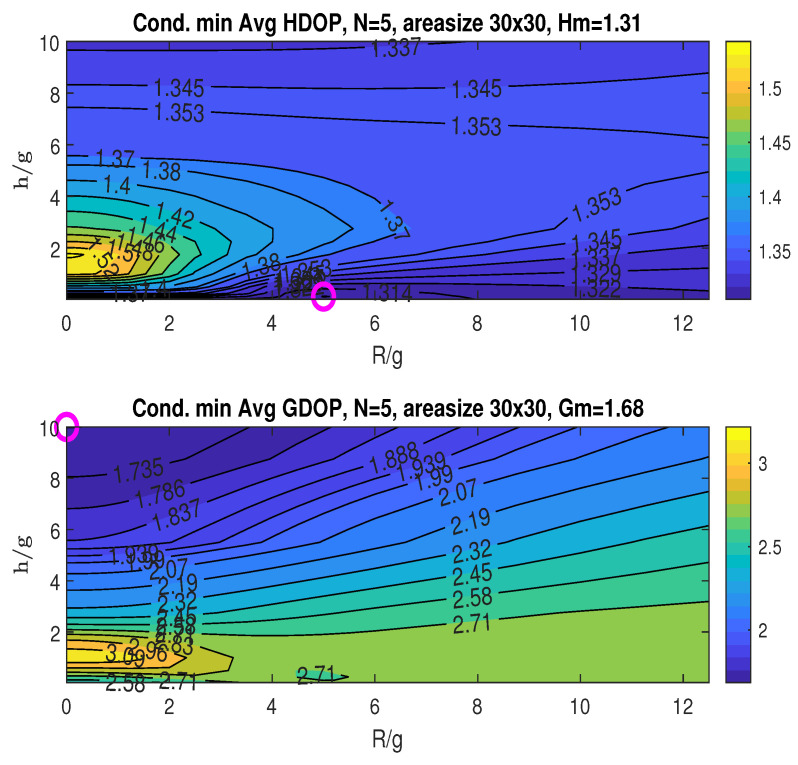
Conditional minimum averaged HDOP and GDOP over area (30×30) with four stationary nodes.

The top graph in Figure 10 shows the conditional minimum averaged HDOP, minimized with respect to the angle β. The minimum HDOP across the plotted ranges of R/g and h/g is $H_{m}=1.31$, as stated in the title. This minimum occurs at $\left(R_{oH}/g,h_{oH}/g\right)=\left(5,0.125\right)$, indicated by the “o” marker. The bottom graph displays the conditional minimum averaged GDOP, also minimized over β. The minimum GDOP across the R/g and h/g ranges is $G_{m}=1.68$, achieved at $\left(R_{oG}/g,h_{oG}/g\right)=\left(0,10\right)$, as marked by “o” in the plot.

**Figure 11:**
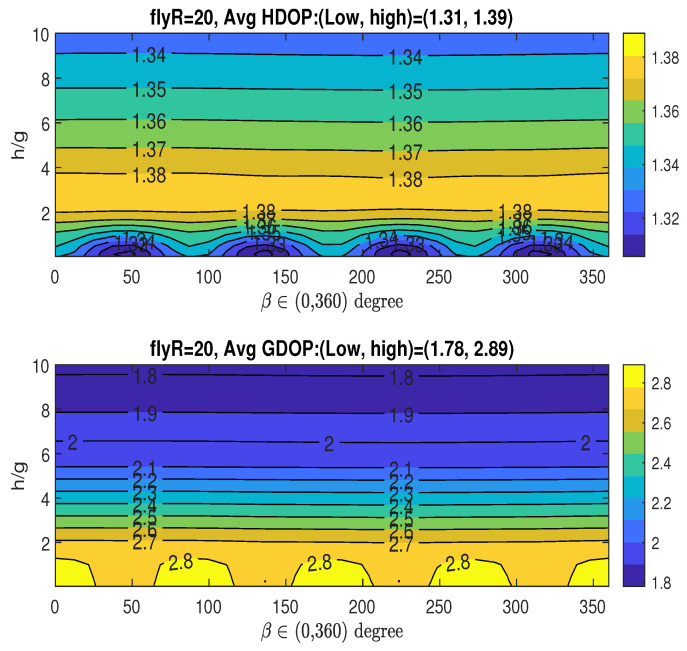
Averaged HDOP and GDOP over area (30×30) with four stationary nodes.

Given $R_{oH}/g=5$, which yields the minimum averaged HDOP ($H_{m}$), the top graph in Figure 11 illustrates the averaged HDOP as a function of the angle $\beta \in (0,360^{\circ })$ and the ratio h/g. The HDOP values range from 1.31 to 1.39, as indicated in the title. The figure suggests that the optimal angle β can be chosen near the following values: $\beta_{oH}\in \left(50^{\circ },136^{\circ },224^{\circ },310^{\circ }\right)$. Recalling the XY-projected angles for the four fixed access nodes in coordinates (13), $\alpha_{i}=\frac{2\pi }{4}\left(i-1\right)$ for i=0,1,2,3, which are $0^{\circ },90^{\circ },180^{\circ }$, and $270^{\circ }$, it is observed that the optimal angle $\beta_{oH}$ is close to $\alpha_{i}+45^{\circ }$.

#### 6.2.2. Area Size (40×40)

**Figure 12:**
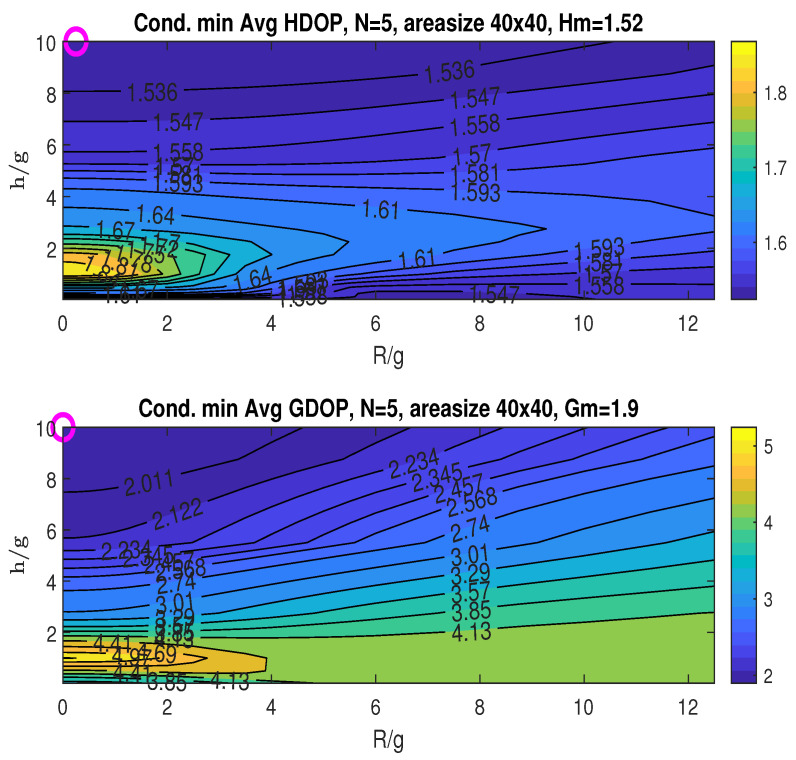
Conditional minimum average HDOP and GDOP over area (40×40) with four stationary nodes.

The conditional minimum averaged HDOP and GDOP, minimized over the angle β, are depicted in Figure 12. The minimum HDOP attained is $H_{m}=1.52$, occurring at $\left(R_{oH}/g,h_{oH}/g\right)=\left(0.25,10\right)$, as indicated by the “o” marker in the plot. The minimum GDOP achieved is $G_{m}=1.9$, found at $\left(R_{oG}/g,h_{oG}/g\right)=\left(0.01,10\right)$. For this considered area size, the minimum achievable averaged HDOP and GDOP are both higher compared to those obtained for the (30×30) size.

**Figure 13:**
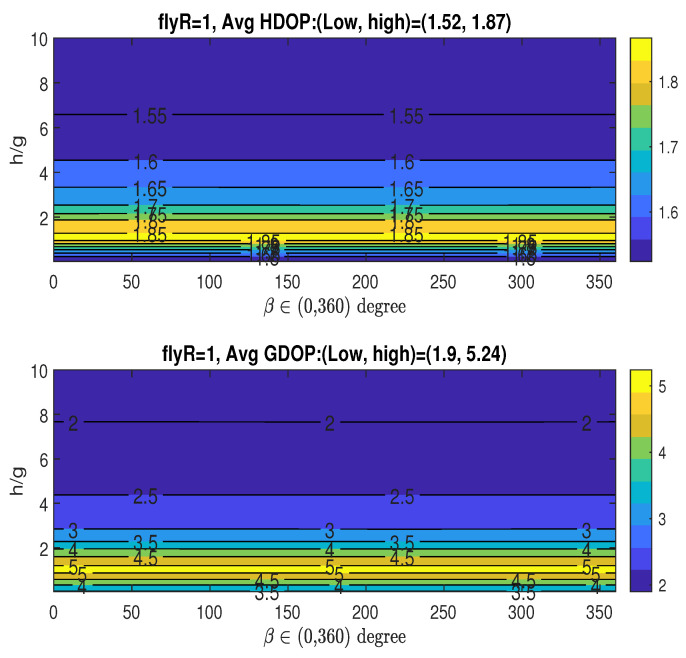
Averaged HDOP and GDOP over area (40×40) with four stationary nodes.

Given that both $R_{oH}$ and $R_{oG}$ have small and similar values, we use $R_{oH}/g=0.25$, which achieves the minimum averaged HDOP ($H_{m}$) for the 2D plotting in Figure 13, which displays the average HDOP and GDOP as functions of β and the ratio h/g. The plots suggest that achieving both $H_{m}$ and the minimum averaged GDOP ($G_{m}$) simultaneously is feasible by choosing a small radius $\overset{˘}{R}/g$ within 0∼0.25, a relatively higher height $\overset{ˇ}{h}/g=10$, and any angle $\overset{˘}{\beta }$ in the range of $0^{\circ }$ to $360^{\circ }$. The specific value of β has little effect on the resulting averaged GDOP and HDOP.

### 6.3. Sensitivity of $H_{m}$ and $G_{m}$ with the Number of Access Nodes

**Figure 14:**
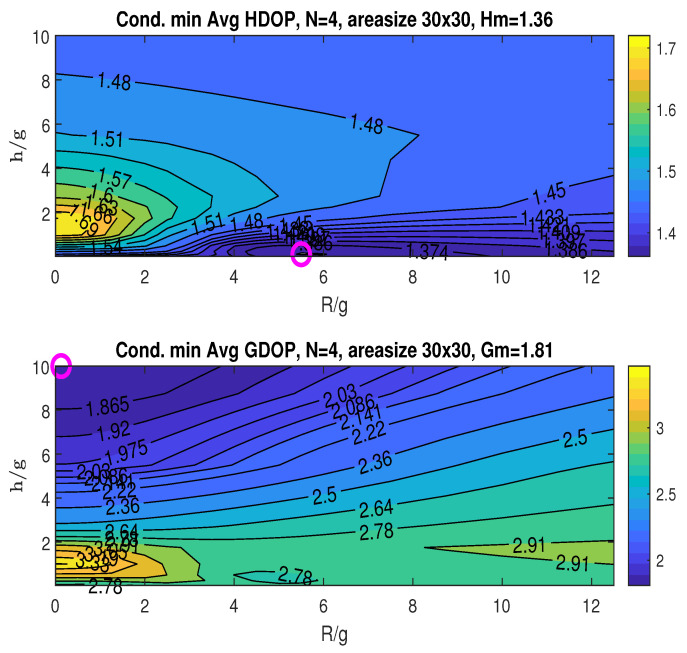
Conditional minimum average HDOP and GDOP over area (30×30) with three stationary nodes.

**Figure 15:**
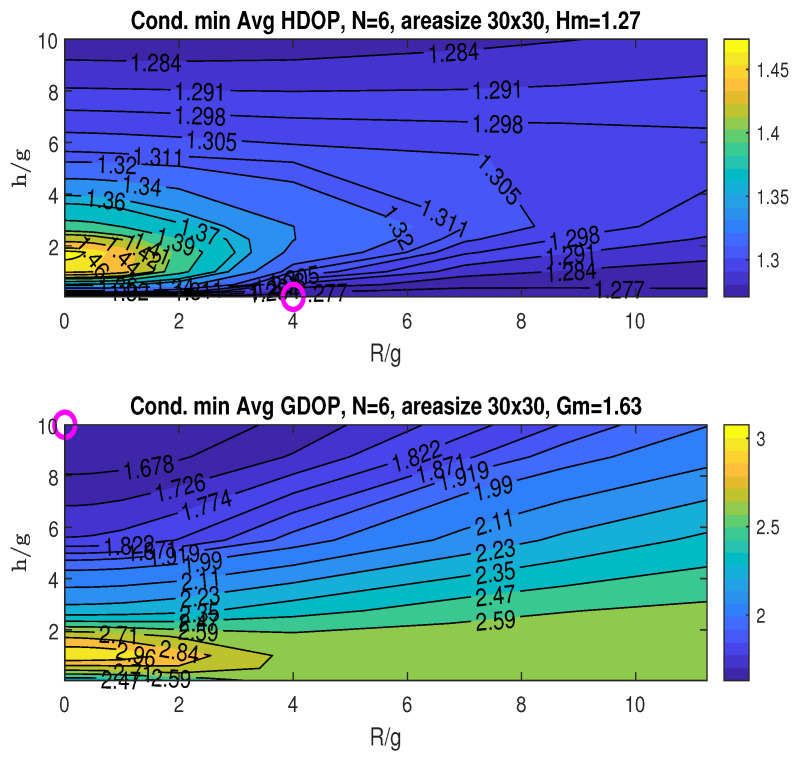
Conditional minimum average HDOP and GDOP over area (30×30) with five stationary nodes.

**Figure 16:**
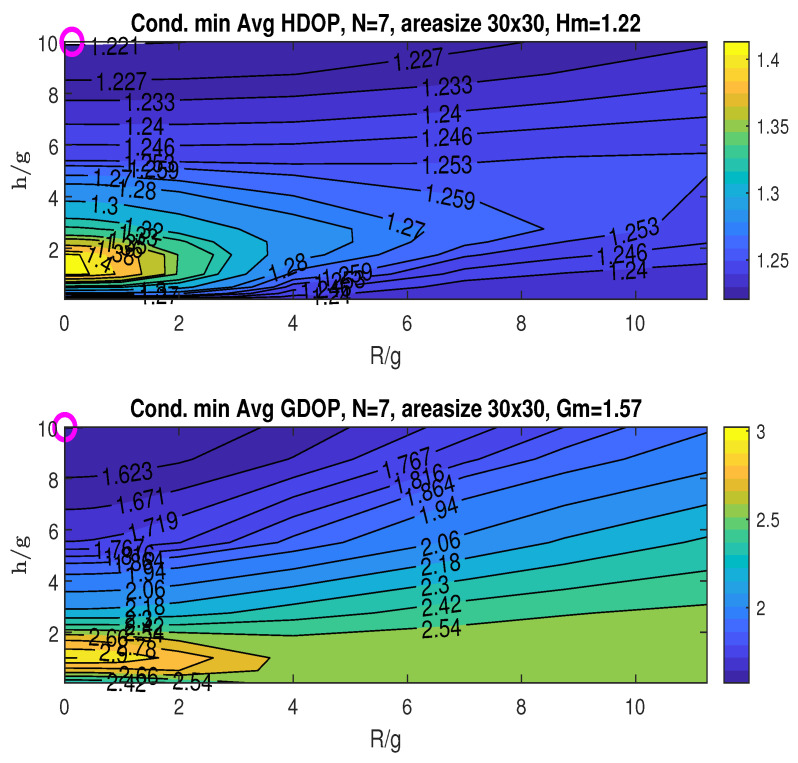
Conditional minimum average HDOP and GDOP over area (30×30) with six stationary nodes.

This simulation investigates how the number of access nodes (*N*) influences the minimum averaged HDOP and GDOP, namely $H_{m}$ and $G_{m}$. A constant area size of 30×30 is maintained for *N* ranging from four to seven. For each *N*, the placement of the stationary nodes (correspondingly three to six) is confined to a cone with the opening angle $\theta_{2}$ chosen by the zenith-horizon placement to minimize GDOP at the origin. The resulting $H_{m}$ and $G_{m}$ values are presented in Figure 10 and Figure 14, Figure 15 and Figure 16, where the minimums are identified by “o” markers and detailed in the figure titles. A summary is provided in Table 1. Increasing the number of access nodes leads to a reduction in both $H_{m}$ and $G_{m}$. However, achieving these minimums concurrently might not always be possible, implying a potential trade-off depending on the application’s specific needs.

### 6.4. Robustness to Misalignment in the Stationary Nodes

In this simulation, we examine the robustness of the proposed algorithm to the misalignment in the stationary nodes. To assess the impact of misalignment in stationary access nodes, the simulation considers errors in the projected XY-plane angles $\alpha_{i}$ (i=1,2,3). The ideal angles are $0^{\circ }$, $120^{\circ }$, and $240^{\circ }$. We analyze two scenarios with perturbed angles ${\tilde{\alpha }}_{i}$:

$$\begin{array}{cc} \mathrm{Case}\;1:\;{\tilde{\alpha }}_{1} & =30^{\circ },\;{\tilde{\alpha }}_{2}=120^{\circ },\;{\tilde{\alpha }}_{3}=255^{\circ }\\ \mathrm{Case}\;2:\;{\tilde{\alpha }}_{1} & =30^{\circ },\;{\tilde{\alpha }}_{2}=120^{\circ },\;{\tilde{\alpha }}_{3}=255^{\circ } \end{array}$$

**Figure 17:**
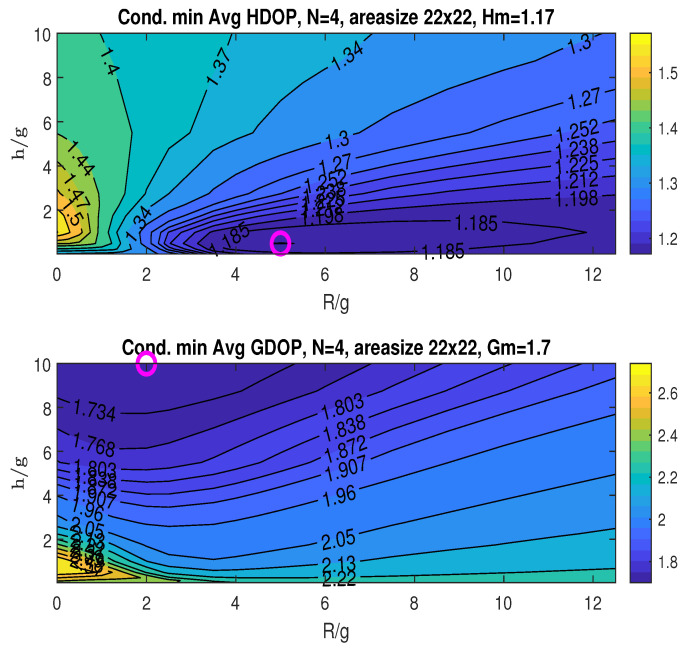
Conditional minimum averaged HDOP and GDOP over a (22×22) area with three stationary nodes under misalignment case 1: ${\tilde{\alpha }}_{1}=\alpha_{1}$, ${\tilde{\alpha }}_{2}=100^{\circ }=\alpha_{2}-20^{\circ }$, and ${\tilde{\alpha }}_{3}=245^{\circ }=\alpha_{3}+15^{\circ }$.

**Figure 18:**
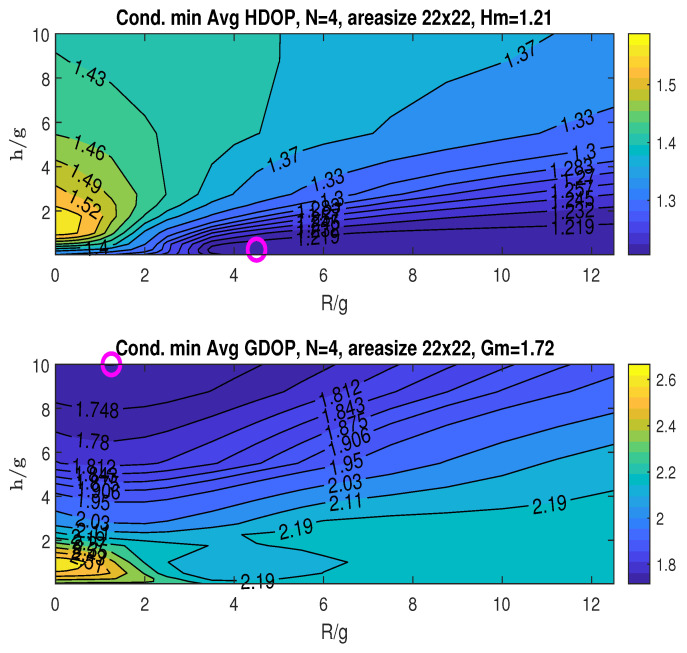
Conditional minimum averaged HDOP and GDOP over a (22×22) area with three stationary nodes under misalignment case 1: ${\tilde{\alpha }}_{1}=60^{\circ }=\alpha_{1}+30^{\circ }$, ${\tilde{\alpha }}_{2}=120^{\circ }=\alpha_{2}$, and ${\tilde{\alpha }}_{3}=245^{\circ }=\alpha_{3}+15^{\circ }$.

From the conditional minimum of the average HDOP and GDOP in Figure 17 and Figure 18, we observe the minimum averaged HDOP ($H_{m}=1.17,1.21$) and GDOP ($G_{m}=1.7,1.72$) under misalignments (marked “o”; details in the figure titles). In contrast, the perfectly aligned scenario in Figure 7 yields $H_{m}=1.23$ at $\left(R_{oH}/g,h_{oH}/g\right)=\left(4,0.125\right)$ and $G_{m}=1.69$ at $\left(R_{oG}/g,h_{oG}/g\right)=\left(0.5,10\right)$.

The results indicate a slight degradation in the minimum averaged GDOP for both misalignment cases. Additionally, both cases exhibit higher worst-case minimum averaged HDOP and GDOP. Interestingly, the minimum averaged HDOP improves under misalignment, albeit achieved at a different UAV position compared to the perfectly aligned case. These findings suggest that the proposed strategy maintains a good degree of robustness against stationary node misalignment.

Table 2 summarizes the detailed values for misalignment Cases 1 and 2, where $H_{\mathrm{wcm}}$ and $G_{\mathrm{wcm}}$ denote the worst conditional mean HDOP and GDOP, respectively.

## 7. Conclusions

This paper presents a UAV-assisted localization approach where a UAV flies in cylindrical paths at different altitudes and radii around the z-axis, augmenting a set of stationary access nodes arranged on a cone. The cone’s opening angle is set based on the zenith-horizon configuration to minimize GDOP at the origin. We investigate a numerical algorithm to find the best measurement points along the UAV’s flight path to minimize the average HDOP and/or GDOP across a defined area where the target node might be located.

Simulation results show that the ideal UAV positions depend on factors such as the total number of access nodes, the elevation and cone angle of the stationary nodes, and the size of the area containing the unknown node. Notably, the optimal UAV position for minimizing average HDOP usually differs from that for minimizing average GDOP, indicating that a single UAV path cannot simultaneously minimize both. If an application focuses on only one DOP metric (HDOP or GDOP), for fewer access nodes, the UAV’s XY-angle can be chosen as a $60^{\circ }$ phase difference compared to the stationary nodes’ XY-angles. However, for more access nodes, the UAV’s XY-angle seems to have a minimal effect on the minimum HDOP or GDOP. In some setups, a compromise between HDOP and GDOP is achievable given acceptable tolerance levels. Furthermore, increasing the number of access nodes generally reduces the minimum HDOP and GDOP values, although achieving these minimums requires careful selection of the UAV’s flight paths.

Since the GDOP metric is derived assuming measurements are only affected by noise, the proposed strategy will not be effective if measurements are unavailable due to signal blockage by severe fading or interference. Furthermore, the proposed numerical solution relies on the UAV’s perfect adherence to the defined trajectories.

## Figures and Tables

**Table 1:** Summary of values for *N*, $H_{m}$, and $G_{m}$ from Figure 10 and Figure 14, Figure 15 and Figure 16.

Total Number of Access Nodes *N*	Min Averaged HDOP $H_{m}$	Min Averaged GDOP $G_{m}$
4	1.36	1.81
5	1.31	1.68
6	1.27	1.63
7	1.22	1.57

**Table 2:** Summary of values for $H_{m},G_{m}$ in perfect alignment and misalignment cases 1 and 2.

	Perfect Alignment	Misalignment Case 1	Misalignment Case 2
$H_{m}$	1.23	1.17	1.21
$H_{\mathrm{wcm}}$	1.5	1.56	1.58
$\left(R_{oH}/g,h_{oH}/g\right)$	(4, 0.125)	(5, 0.2)	(4.5, 0.1)
$G_{m}$	1.69	1.7	1.72
$G_{\mathrm{wcm}}$	2.4	2.67	2.65
$\left(R_{oG}/g,h_{oG}/g\right)$	(0.5, 10)	(2, 10)	(1.5, 10)

## Data Availability

Data are contained within the article.

## Appendix A. Proof for (10)

The geometric matrix in (3) at the origin is given by

(A1) $$\begin{array}{c} A=\left(\begin{array}{ccc} e_{x} & e_{y} & e_{z} \end{array}\right)=\left(\begin{array}{ccc} \frac{x_{0}}{\rho_{0}} & \frac{y_{0}}{\rho_{0}} & \frac{z_{0}}{\rho_{0}}\\ \frac{x_{1}}{\rho_{1}} & \frac{y_{1}}{\rho_{1}} & \frac{z_{1}}{\rho_{1}}\\ \frac{x_{2}}{\rho_{2}} & \frac{y_{2}}{\rho_{2}} & \frac{z_{2}}{\rho_{2}}\\ \vdots & \vdots & \vdots \\ \frac{x_{N-1}}{\rho_{N-1}} & \frac{y_{N-1}}{\rho_{N-1}} & \frac{z_{N-1}}{\rho_{N-1}} \end{array}\right). \end{array}$$

where the coordinates of access nodes are

(A2) $$\begin{array}{c} \left(x_{i},\;y_{i},\;z_{i}\right)=\left(\rho_{i}\sin\theta_{i}\cos\alpha_{i},\rho_{i}\sin_{i}\theta_{2}\sin\alpha_{i},\rho_{i}\cos_{i}\right),i=0,1,2,\ldots ,N-1 \end{array}$$

We have

$$A^{T}A=\left(\begin{array}{ccc} \sum_{i=0}^{N-1}\frac{x_{i}^{2}}{\rho_{i}^{2}} & \sum_{i=0}^{N-1}\frac{x_{i}y_{i}}{\rho_{i}^{2}} & \sum_{i=0}^{N-1}\frac{x_{i}z_{i}}{\rho_{i}^{2}}\\ \sum_{i=0}^{N-1}\frac{x_{i}y_{i}}{\rho_{i}^{2}} & \sum_{i=0}^{N-1}\frac{y_{i}^{2}}{\rho_{i}^{2}} & \sum_{i=0}^{N-1}\frac{y_{i}z_{i}}{\rho_{i}^{2}}\\ \sum_{i=0}^{N-1}\frac{x_{i}z_{i}}{\rho_{i}^{2}} & \sum_{i=0}^{N-1}\frac{y_{i}z_{i}}{\rho_{i}^{2}} & \sum_{i=0}^{N-1}\frac{z_{i}^{2}}{\rho_{i}^{2}} \end{array}\right).$$

Inserting the coordinates of access nodes, we have $\mathrm{tr}\left(A^{T}A\right)=\sum_{i=0}^{N-1}\frac{x_{i}^{2}+y_{i}^{2}+z_{i}^{2}}{\rho_{i}^{2}}=N$. Denote the eigen values for matrix $A^{T}A$ as $\lambda_{i},i=1,2,3.$ The minimum GDOP solution is formulated as follows:

$$\mathrm{tr}\left(A^{T}A\right)=\lambda_{1}+\lambda_{2}+\lambda_{3}=N$$

$$\mathrm{GDOP}=\sqrt{\mathrm{tr}({\left(A^{T}A\right)}^{-1})}=\sqrt{\frac{1}{\lambda_{1}}+\frac{1}{\lambda_{2}}+\frac{1}{\lambda_{3}}}$$

Consequently, the problem of minimizing GDOP can be formulated as minimizing the function $\sum_{i=1}^{3}\frac{1}{\lambda_{i}}$ subject to the constraint $\sum_{i=1}^{3}\lambda_{i}=N$. By the Arithmetic Mean–Harmonic Mean (AM-HM) inequality, the sum of reciprocals is minimized when all $\lambda_{i}$ are equal:

$$\lambda_{1}=\lambda_{2}=\lambda_{3}=\frac{N}{3}\Rightarrow A^{T}A=\frac{N}{3}I$$

□
